# An Intervention Mapping Approach to Developing a Stroke Literacy Video for Recent Stroke Survivors: Development and Usability Study

**DOI:** 10.2196/31903

**Published:** 2023-01-04

**Authors:** Mary Carter Denny, Andrea Ancer Leal, Tahani Casameni Montiel, Keona J Wynne, Gabrielle Edquilang, Kim Yen Thi Vu, Farhaan Vahidy, Sean I Savitz, Jennifer ES Beauchamp, Anjail Sharrief

**Affiliations:** 1 Department of Neurology Georgetown University Medical Center MedStar Health Washington, DC, DC United States; 2 Department of Research Cizik School of Nursing at UTHealth Houston, TX United States; 3 Department of Social and Behavioral Sciences Harvard T.H. Chan School of Public Health Harvard University Boston, MA United States; 4 Memorial Hermann – Texas Medical Center Houston, TX United States; 5 Center for Outcomes Research Houston Methodist Houston, TX United States; 6 Department of Population Health Sciences Weill Cornell Medical School New York, NY United States; 7 Houston Methodist Neurological Institute Houston Methodist Houston, TX United States; 8 Department of Neurology McGovern Medical School UTHealth Houston, TX United States; 9 UTHealth Institute for Stroke and Cerebrovascular Disease Houston, TX United States

**Keywords:** stroke, stroke prevention, health literacy, stroke literacy, patient education, transition of care, risk factors, cardiac, digital health

## Abstract

**Background:**

Most vascular events after stroke may be prevented by modifying vascular risk factors through medical and behavioral interventions. Stroke literacy—an understanding of stroke symptoms, risk factors, and treatment—likely contributes to vascular risk factor control and in turn stroke recurrence risk. Stroke literacy is the lowest among adults belonging to racial and ethnic minority populations in the United States. Video-based interventions targeting stroke literacy may help acute stroke survivors understand stroke and subsequently reduce the risk of stroke recurrence. However, the failure of prior stroke literacy interventions may be due in part to the fact that the interventions were not theory-driven. Intervention mapping (IM) provides a framework for use in the development, implementation, and evaluation of evidence-informed, health-related interventions.

**Objective:**

We aimed to develop a video-based educational intervention to improve stroke literacy in hospitalized patients with acute stroke.

**Methods:**

The 6-step iterative process of IM was used to develop a video-based educational intervention and related implementation and evaluation plans. The six steps included a needs assessment, the identification of outcomes and change objectives, the selection of theory- and video-based intervention methods and practical applications, the development of a video-based stroke educational intervention, plans for implementation, and evaluation strategies.

**Results:**

A 5-minute video-based educational intervention was developed. The IM approach led to successful intervention development by emphasizing stakeholder involvement, generation and adoption, and information retainment in the planning phase of the intervention. A planned approach to video adoption, implementation, and evaluation was also developed.

**Conclusions:**

An IM approach guided the development of a 5-minute video-based educational intervention to promote stroke literacy among acute stroke survivors. Future studies are needed to assess the use of technology and digital media to support widespread access and participation in video-based health literacy interventions for populations with acute and chronic stroke. Studies are needed to assess the impact of video-based educational interventions that are paired with stroke systems of care optimization to reduce the risk of stroke recurrence. Furthermore, studies on culturally and linguistically sensitive video-based stroke literacy interventions are needed to address known racial and ethnic disparities in stroke literacy.

**International Registered Report Identifier (IRRID):**

RR2-10.1371/journal.pone.0171952

## Introduction

There are more than 7 million stroke survivors in the United States and 80 million worldwide [1]. Mortality from stroke has steadily declined over the past 10 years, which is due in part to advances in acute stroke treatments and the establishment of comprehensive stroke centers [2]. However, the incidence of stroke continues to rise, which is driven primarily by an aging population [3]. The total annual cost of stroke in the United States, including direct medical costs and indirectly lost productivity, is estimated to be US $120 billion currently, and it is projected to double to US $240.7 billion by 2030 [4]. Given the large public health impact of stroke, Healthy People 2030 [5] includes goals for increasing the proportion of people who receive acute stroke treatment and decreasing stroke-related mortality rates. In the 2017 National Health Interview Survey, only 67.5% of adults over 20 years old were aware of the common signs and symptoms of stroke and the necessity of activating emergency medical services (EMS) [6]. Among young adults, 28.9% were unable to name 5 stroke symptoms [7]. Acute stroke treatments are time-dependent; therefore, the early recognition of the common stroke symptoms and activation of EMS are crucial to decreasing disability and mortality from stroke.

Of the 795,000 strokes that occur in the United States annually, 185,000, or nearly 25%, occur in patients with a history of prior stroke [8]. Up to 80% of vascular events after stroke may be prevented by modifying vascular risk factors through medical and behavioral interventions; however, up to 40% of patients experience a second stroke within 10 years [9,10]. Multiple variables, including stroke literacy, likely contribute to vascular risk factor control and stroke recurrence risk in turn. Stroke literacy, which is an understanding of stroke signs and symptoms, risk factors, and treatment, is vital for secondary stroke prevention campaigns [11]. Stroke survivors and their family members reported unmet educational needs, including an understanding of the causes of strokes and how to prevent future strokes [12,13]. The Joint Commission, the principal accreditation organization that certifies hospital stroke services in the United States, has recognized the importance of addressing this stroke literacy gap and requires that all stroke survivors and their families receive stroke education [14]. However, the format, language, and content of this mandatory stroke education is not defined clearly. Studies suggest that Hispanic groups are less aware of stroke signs and symptoms, opportunities for acute stroke treatment, and the crucial time from stroke onset to treatment [6,7,15-17]. Data from the 2019 census in the United States showed that 18.4% of the country’s population identified as Hispanic [18]. Despite the large number of Hispanic adults at risk for a stroke, there are limited stroke literacy educational interventions tailored for Spanish-speaking populations [19].

Video-based educational interventions have demonstrated higher efficacy in increasing knowledge and modifying health behaviors than written educational materials for other chronic diseases [20]. Video-based interventions targeting stroke literacy may help stroke survivors understand stroke and lead to a reduction in stroke recurrence. However, many prior stroke literacy interventions have been unsuccessful in achieving sustained knowledge acquisition and stroke-related behavior change [21]. The failure of these stroke literacy interventions may be in part because although they were pragmatic “commonsense interventions,” they were neither theory-driven interventions nor evidence-based interventions [22,23]. Being explicit in the theory used to develop health interventions mitigates the unintended consequence of differential uptake of the intervention, whereby more educated individuals will gain more from the intervention than less educated individuals, who often carry a larger disease burden [24]. The 2021 secondary stroke prevention guidelines from the American Heart Association/American Stroke Association emphasize that “Changing patient behaviors such as diet, exercise, and medication compliance requires more than just simple advice or a brochure from their physician. Programs that use theoretical models of behavior change, proven techniques, and multidisciplinary support are needed” [25].

Intervention mapping (IM) is “a planning approach that is based on the importance of developing theory- and evidence-informed programs, taking an ecological approach and intervening in health problems and community participation” [22]. IM provides a framework for health promoters to use in the development, implementation, and evaluation of health-related interventions. An IM approach is an iterative process comprising the following six steps: (1) needs assessment, (2) identification of outcomes and change objectives, (3) selection of theory-based intervention methods and practical applications, (4) development of the intervention program, (5) implementation, and (6) evaluation. Therefore, the primary aim of this study was to develop a video-based educational intervention, using an IM approach, for stroke survivors in the acute hospital setting.

## Methods

### Ethics Approval

This study was approved by the university’s institutional review board (HSC-MS-14-0931). All study participants or their legally authorized representatives provided written informed consent prior to the initiation of participant-related study activities, such as the implementation and evaluation of the video-based educational intervention.

### Intervention Developed Using the IM Approach

#### Overview of the IM Approach

The video-based educational intervention was developed using the 6-step IM approach, which is outlined in Textbox 1.

**Intervention mapping steps and descriptions (reprinted with permission from Bartholomew et al [26]).**

**Step 1: Needs assessment**

- Create a planning committee
- Determine needs
- Assess community knowledge and attitude

**Step 2: Change objectives**

- Determine outcomes for behavioral and environmental change

**Step 3: Theoretical methods and practical applications**

- Develop program intervention ideas with the aid of a planning committee
- Verify that applications are aligned with previously identified change objectives

**Step 4: Intervention program**

- Create the program
- Pretest the program

**Step 5: Adoption and implementation**

- Determine the procedures and protocols necessary for adoption

**Step 6: Evaluation**

- Assess program concept and design
- Review logic model
- Write evaluation questions

#### Step 1: Literature Review and Needs Assessment Involving Major Stakeholders

A literature review on the educational needs of stroke survivors and previous in-hospital and posthospitalization stroke educational interventions was conducted. Moreover, published studies on stroke educational interventions and health behavior change theories that would be most suitable for the development of a video-based educational intervention for recent stroke survivors were identified. The literature review was conducted using PubMed and other available library resources, using the search terms “educational video,” “health literacy,” “stroke education,” “stroke literacy,” “stroke prevention, “educational intervention,” and “transitions of care.” The search was restricted to publications in English only.

An interview-based needs assessment was conducted at a large academic medical center in Southeast Texas, involving approximately 50 acute stroke survivors and their family members. Our multidisciplinary stroke care team, including rehabilitation therapists, a social worker trained as a transitions of care coordinator, case managers, stroke-trained nurses, and stroke physicians, was also interviewed to identify common concerns and stroke knowledge gaps. The educational needs of stroke survivors and their family members were based on the most frequently asked questions and topics during the monthly hospital-based stroke support group. Medical and nursing staff directly involved in the care of stroke survivors were asked about the perceived educational needs of the patients and their family members in terms of the educational gaps they commonly encountered. Additionally, the study team reviewed call logs compiled by the nurse in charge of the stroke unit to identify 7-day postdischarge telephone calls to stroke survivors and their caregivers and common stroke knowledge needs. Finally, 2 stroke physicians identified common questions raised by stroke survivors and their caregivers at outpatient follow-up visits.

#### Step 2: Identification of Outcomes, Determinants, and Change Objectives

Using the information gathered in the literature review and needs assessment, several prominent gaps in stroke knowledge and outcomes in the population with stroke were identified. Short-, medium-, and long-term outcomes were identified, using a logic model, to develop a structure for determining how a video-based educational intervention would fit into the larger goal of stroke sign and symptom recognition as well as secondary stroke prevention.

#### Step 3: Selection of Theory-Based Intervention Methods and Practical Applications

The McGuire communication persuasion matrix (CPM) theory [26-28] was selected to identify theoretical methods, as it was the most relevant one for this population of adult learners. The McGuire CPM theory uses 7 principles to identify theoretical methods and anticipate how differences in the character, personality, and rationale of individuals will affect their adoption of a health promotion campaign [26-29]. The McGuire CPM has been adapted and updated by public health experts to include elements from the Bandura social cognitive theory (attitude, social influences, and self-efficacy) [26].

#### Step 4: Development of the Video Intervention and Questionnaire

The most complex steps were the following: (1) developing the video-based educational intervention with a video script at a sixth-grade reading level, (2) creating a novel pre- and postintervention questionnaire, (3) engaging multiple health care team members in filming the video, and (4) obtaining hospital administrative approvals.

Members of the stroke care team were engaged to film the video in a question-and-answer format, followed by the editing of the video. The overall development of the video-based educational intervention and questionnaire involved iterative processes that took place over a 6-month period. A novel 10-item questionnaire was developed by the principal investigator, with inputs from the multidisciplinary stroke team based on the major gaps identified in step 1. Items 1 to 8 assess stroke knowledge, item 9 evaluates self-efficacy in identifying stroke symptoms, and item 10 measures satisfaction with stroke education (Multimedia Appendix 1).

Although not a part of the original study, in 2020, a team of diverse Spanish-speaking research team members collectively translated the video content into Spanish. The initial draft of the Spanish transcript was created by 1 member, and the other 2 members reviewed it independently for accuracy. Any disagreements in word choice were reviewed and resolved, using Spanish grammar resources. The final translation of the transcript was added in the video as Spanish subtitles.

#### Step 5: Generation of the Adoption and Implementation Plan

An implementation plan was developed by using the IM approach. Key stakeholders, including stroke nurses, social workers, and stroke physicians, came together to develop a plan to implement the video intervention for stroke survivors once they were medically stable. We tried showing the video on hospital room televisions, computers on wheels, and a bedside laptop. Study team members developed a plan to screen patients for participation in the educational video intervention study during daily multidisciplinary rounds and identify them once they were nearing hospital discharge. All potential study participants had already received standard verbal and written stroke education from their bedside nurses, physicians, and rehabilitation therapists, as appropriate.

#### Step 6: Generation of an Evaluation Plan

An evaluation plan was generated with the stroke care team.

## Results

### Step 1: Literature Review and Needs Assessment Involving Major Stakeholders

The literature review yielded 10 studies [11,21,30-37] focusing on stroke literacy. The need for theory-based interventions designed with materials that are culturally tailored and are at the appropriate health literacy level was emphasized [21,24]. Prior stroke education studies had primarily used written materials as a teaching modality, and few had focused on recent stroke survivors in their transition of care as they left the acute hospital setting [11,31-33].

We identified stroke knowledge gaps from our stroke support group, which were raised by recent stroke survivors and their caregivers, including common questions like “what is a stroke” and “what happens after I leave the hospital?” Using the information gathered from multiple stakeholders, we identified the most common stroke knowledge gaps—the definition of stroke, warning signs and symptoms of stroke, risk factors for stroke, the activation of EMS, and follow-up after discharge. These stroke knowledge gaps were in line with the stroke education mandate set forth by the Joint Commission as part of stroke center certification [14,38].

### Step 2: Identification of Outcomes and Change Objectives

Several prominent gaps in stroke knowledge among the population with stroke were identified, including the definition of stroke, the recognition of stroke signs and symptoms, the importance of activating EMS if stroke is suspected, the management of risk factors for stroke prevention, and the importance of follow-up care. Additionally, stroke survivor satisfaction with stroke education was identified as an important topic for formative outcome data to the major stakeholders.

Short-term outcomes were identified that assessed the impact of the educational video intervention on stroke knowledge acquisition, self-efficacy in identifying stroke symptoms, and satisfaction among recent stroke survivors prior to hospital discharge. The medium-term outcomes of self-efficacy in identifying stroke symptoms and activating 911 (EMS) were assessed. Additionally, the keeping of outpatient follow-up appointments, home blood pressure monitoring, and adherence to medications were also identified. These identified short-term outcomes may be necessary, although not sufficient alone, to affect the identified medium-term outcomes of attending follow-up appointments and monitoring blood pressure at home. Long-term outcomes were not measured in this pilot study (Textbox 2).

**Logic model details from step 2.**

**Input**

- Staff: 1 to 2 program staff members to deliver video and answer questions
- Materials: 5-minute educational video shown on bedside laptop

**Activities**

- One 5-minute video covering the following:
  Recognition of stroke symptoms
  Importance of calling 911 for suspected stroke
  Control of stroke risk factors
  Importance of outpatient follow-up visits
- Facilitators will answer any questions and clarify information for participants

**Outputs**

- Number of staff members trained to provide video to patients and family members
- Number of patients and family members who participate in the video session

**Short-term outcomes**

- Increase at-risk participants’ knowledge of the following:
  Stroke symptoms
  Importance of calling 911
  Control of stroke risk factors
  Importance of outpatient follow-up visits
- Improve stroke knowledge in participants, so that they better understand the following:
  Basic physiology of stroke
  Medical interventions that took place in the acute stroke setting
  How to prevent another stroke in the future
- Increase previous stroke survivors’ and their caregivers’ knowledge of stroke risk factors (ie, high blood pressure, high cholesterol, diabetes, and tobacco use)

**Intermediate outcomes**

- Increase participants’ abilities to recognize stroke symptoms in themselves and others and to take appropriate action (ie, calling 911)
- Improve previous stroke survivors’ ability to modify stroke risk factors, such as the following:
  Keeping 75% of outpatient follow-up visits
  Monitoring blood pressure daily at home
  Adhering to chronic disease medications for at least 80% of the time

**Long-term outcomes**

- Decrease the incidence of stroke (including recurrent stroke) and the morbidity and mortality from stroke in previous stroke survivors

### Step 3: Selection of Theory-Based Methods and Practical Strategies

An IM approach was selected because it uses health behavior theory to plan and develop health promotion interventions and has been successful in prior stroke education interventions [22,39,40]. A video modality for the practical delivery of the educational intervention was chosen, as it has proved more effective than written material in promoting knowledge acquisition and behavior change in several health conditions [20].

### Step 4: Development of the Intervention Program (Making the Video)

A 5-minute educational video with high emphasis on incorporating diagrams and visual materials was created. Specifically, a full-screen animation module simulated both ischemic and hemorrhagic strokes occurring in the brain. The animations were shown after the physicians defined and discussed each type of stroke separately to avoid confusion. Visuals were also used to illustrate the acronym *FAST* (face, arm, speech, and time) with regard to calling 911. Each visual corresponded with each letter of the acronym and appeared one at a time on the screen until the entire acronym was present. A voice-recording explaining this acronym was used to supplement the visual material presented.

The video intervention (Multimedia Appendix 2) also used words and visuals to demonstrate stroke risk factors (eg, eating a greasy hamburger for high cholesterol). The video used visuals to illustrate specific ways to control risk factors (eg, blood sugar monitoring for diabetes). Special emphasis was placed on hypertension—the most major stroke risk factor—with a demonstration of how to monitor blood pressure at home. Finally, the use of imagery and modeling allowed for visual and physical cues to be created. As an example of modeling, the use of a blood pressure cuff (video modeling) by a stroke survivor was shown when hypertension was discussed, creating a visual cue. Care was taken to ensure that a diverse cast (patients and health care professionals alike) was included in the video, as it has been proven that the effectiveness of video modeling increases when the cast is ethnically consistent with the target population [20]. The stroke literacy topics covered in the video included what is a stroke, the recognition of stroke symptoms, the activating of EMS by calling 911, stroke risk factors, stroke prevention, transition to rehabilitation, and the importance of outpatient follow-up.

In 2020, based on our initial results, epidemiologic data, and the geographic location of our institution in Southeast Texas, we decided to translate the contents of the video into Spanish, checking for linguistic accuracy. We are currently investigating if our results hold when applied to a broader Spanish-speaking population.

The questionnaire included 8 questions related to the knowledge of stroke, stroke sign and symptom recognition, risk factors for stroke, and stroke prevention; 1 question regarding self-efficacy in recognizing stroke symptoms; and 1 question about patient satisfaction with stroke education (Multimedia Appendix 1).

### Step 5: Generation of the Adoption and Implementation Plan

Hospital computers on wheels were found to often be in use by providers and would not be consistently accessible to study staff. The logistical challenge of having a large video file to upload onto a hospital-networked computer was also identified. As such, we considered showing the video on the television in the stroke survivor’s hospital room, but we soon realized that if the video was uploaded onto the hospital content channel, we would not have control over how many times patients with stroke viewed the video during their hospital stay, which could alter the study results. Therefore, we found it most feasible to show the video to stroke survivors on a bedside laptop that we could adjust according to their bed position and line of sight. We found that, owing to the ambient hospital noise (ie, monitors beeping and staff going in and out of the room), several patients required external speakers with the laptop to provide adequate audio volume.

Prior to enrolling the first study participant, each member of the study team would be trained on performing the procedures of obtaining informed consent, administering the 10-item questionnaire in person and by telephone, showing the 5-minute educational video, and answering common questions from participants and their informal caregivers (eg, spouses and family members).

The 5-minute stroke educational video was implemented for use in the hospital before the discharge of recent stroke survivors at the Memorial Hermann Hospital - Texas Medical Center (MHH-TMC). The MHH-TMC is a high-volume, comprehensive stroke center located in Houston. The enrollment period for the study was from March to June 2015.

All of the participants had the opportunity to complete the 10-item questionnaire by using a pen and paper, per their convenience. For study participants with visual impairment, illiteracy, neglect, or reading impairment due to stroke, the study personnel read the questionnaire aloud. For participants with dominant hand weakness or impaired dexterity, study personnel could circle the answer choice they provided verbally. In cases where the participants could not complete any items on the questionnaire due to somnolence, cognitive impairment, aphasia, or other reasons, the study personnel checked this box on the questionnaire accordingly. As noted above, the study participants completed the 10-item questionnaire before, immediately after, and 30 days following the viewing of the stroke video.

### Step 6: Generation of an Evaluation Plan

The novel 10-item questionnaire was used to evaluate stroke knowledge before, immediately after, and at 30 days following the viewing of the stroke educational video, and the results were published in a peer-reviewed journal [41].

The external review of the video-based stroke education intervention study was conducted by public health professionals with expertise in health promotion. The review included an evaluation and critique of the study design, implementation, and outcomes. A logic model generated in the initial program description was used as a guide in the external review (Textbox 2).

### Primary Pilot Results

A full account of the pilot findings can be found in the authors’ previously published primary results [41]. In summary, 250 inpatient stroke survivors were screened for participation, 102 were enrolled in the study, and 93 completed the video intervention. The short-term outcomes were stroke literacy and self-efficacy in identifying stroke symptoms. Stroke literacy was measured before, immediately after, and 30 days following the viewing of the stroke educational video, using a 10-item questionnaire. The median stroke knowledge score on the questionnaire before viewing the video was 6 (IQR 4-7) out of 8 and increased to a median score of 7 (IQR 6-8; *P*<.001) after viewing the video. The proportion of participants who were “very satisfied” with their education increased from 49.5% before viewing the video to 74.2% after viewing the video (*P*<.01), and this was also maintained at 30 days (75.4%; *P*<.01). A median stroke knowledge score of 7 (IQR 5-8; *P*=.04) was maintained at 30 days. The proportion of participants who were “very certain” in recognizing stroke symptoms increased from 35.5% before viewing the video to 53.5% after viewing the video, and this was maintained at 30 days (35.5% vs 53.5%, *P*=.01; 35.5% vs 54.4%, *P*=.02) [41].

## Discussion

### Primary Findings

To our knowledge, this remains the first theory-driven study of a video-based stroke literacy intervention, which was developed by using IM, for recent stroke survivors and their families in the acute hospital setting. The development and implementation of this stroke literacy video required input from a multidisciplinary team that included nurses, physicians, social workers, and rehabilitation therapists alongside stroke survivors and their families. The individuals portrayed in the video are diverse in terms of race, ethnicity, sex, and age, which is a notable strength. The stroke education video was designed to be compatible with a wide range of literacy levels and address the most common concerns of stroke survivors and their families in the transition of care from the acute care hospital to rehabilitation centers or homes [42-45]. The clinical team at our hospital continues to receive positive feedback on the video not only from patients but also from their families and caregivers, who often watch the video with them as they prepare for hospital discharge. However, we learned that it takes cooperation from the hospital administration, bedside nurses, and physicians to ensure that the video continues to be shown as part of the services provided in a busy inpatient stroke unit.

As an educational tool, the video format allows stroke survivors and their families to rewind, pause, and rewatch the video while watching it at a speed that is comfortable for them in the privacy of their hospital room. The ability to ask questions after viewing the video along with a health care provider may remove some of the stigma and potential shame of not understanding health information when it is presented initially. The use of the video allowed imagery to be incorporated, which can aid visual learners and solidify concepts for auditory learners. Visual materials have been used in multiple behavior change strategies, but there is a lack of literature focusing on stroke survivors. Unlike text, visuals can be perceived and understood immediately upon their view [28]. It is necessary for the visual materials to incorporate elements that are familiar and personable to the target population [26,28]. Furthermore, visuals should strive to be realistic as opposed to being symbolic, simple with few distracters, illustrate health-promoting activities with visuals, and be used to stimulate interaction [26]. The video also includes text, which is accompanied by images that can demystify some of the hard-to-understand medical language and help patients begin to build a recognition system, as they encounter the same terms in their follow-up care.

### Comparison With Prior Work

Previous studies on stroke messaging via television advertising have shown significant increases in correct responses to warning signs of stroke [46-48]. When 2173 participants in Ohio were asked to report their sources of knowledge, television made up 32% of the cited sources [49]. One study even suggested that the population-level distribution of television campaigns was associated with a significant increase in emergency department visits related to stroke [48]. If media advertising, specifically television or video advertising, can increase the number of people who take emergency action when experiencing stroke symptoms, our video-based educational intervention has important consequences. Informal video presentation platforms, such as YouTube, have been shown to reach over 1 billion users, with many videos related to medical information [50]. Studies have reported that social media and general search engines are some of the most common avenues used to find health-related information [51]. Although misinformation or a lack of scientific relevance is an issue in these informal settings, the outreach could be beneficial and increase the amount of accurate information on such sites [52]. In waiting rooms or physician offices, educational interventions have been shown to increase patient satisfaction, and one television-based health advertisement intervention resulted in a significant increase in knowledge and the intention to seek medical help among patients after they viewed the video in a general practice waiting room [53,54]. This evidence suggests that partnering with care providers in various health care settings to promote the use of our video in waiting rooms could offer a streamlined avenue to stroke survivors and their families, as well as those at risk for stroke.

### Limitations

A key limitation was that the audio of our stroke literacy video was only available in English. Although our team added Spanish subtitles to the English audio in 2020, Spanish-speaking stroke survivors may miss important content if they are slow readers and cannot watch the video at normal play speed. Furthermore, the video may exclude those with no reading ability. Although the inclusion of Spanish subtitles is a step toward providing language-competent video-based stroke education to Spanish-speaking stroke survivors and their families, translating the script and recording the video in various languages, including Spanish, will be important for future studies. The pilot study excluded 23 potential participants because they were not English speakers, and the study inclusion criteria required English fluency [41]. If translated into multiple languages, the video could be further tailored to different cultural groups. For example, healthy food choices from various cuisines could be incorporated.

### Conclusions

We received feedback from patients and families stating that they wanted access to the video to view it again after they went home. A future aim is to make the video easily accessible to stroke survivors and their families after hospital discharge to reinforce key messages and promote behavior changes associated with stroke risk reduction.

We plan to pair the video with a skill-building activity for risk factor self-management, along with subsequent viewings at patients’ follow-up visits in the stroke clinic. One such skill-building activity would be training stroke survivors and their families to perform home blood pressure monitoring. Moreover, in our pilot study, we collected longitudinal data to assess the impact of the video on stroke literacy, self-efficacy, and patient satisfaction at longer-term time points [40]. In a future study, we will assess the impact of the video on both stroke literacy and behavior change outcomes at short- and longer-term time points. A future multicenter study with the video translated into Spanish and other languages has been planned.

## Appendix

Multimedia Appendix 1 Stroke knowledge questionnaire.

Multimedia Appendix 2 Stroke video.

## Abbreviations

CPM: communication persuasion matrix
EMS: emergency medical services
FAST: face, arm, speech, and time
IM: intervention mapping
MHH-TMC: Memorial Hermann Hospital-Texas Medical Center
