# Supplementary material for: An Intervention Mapping Approach to Developing a Stroke Literacy Video for Recent Stroke Survivors: Development and Usability Study
Source: JMIR Form Res. 2023 Jan 4;7:e31903. doi: 10.2196/31903 (PMC9850284; doi:10.2196/31903)
Supplement: Multimedia Appendix 1 [file formative_v7i1e31903_app1.docx]

| **Stroke Knowledge Questionnaire**  **□ Check here if patient unable to complete the survey**  **Date: __________**   1. What is the main medical problem that you were hospitalized for?    1. Ischemic Stroke (Stroke caused by a blocked blood vessel)    2. Hemorrhagic Stroke (Stroke causes by a burst blood vessel)    3. Transient Ischemic Attack (TIA)    4. Seizure    5. I don’t know 2. Where in the body does a stroke occur?    1. Heart    2. Brain    3. Kidneys    4. Lungs    5. I don’t know 3. What causes a stroke?    1. When a blood vessel in your brain gets blocked or bursts and that part of the brain doesn’t work anymore    2. When you sleep on one side of your body and pinch a nerve in your arm or leg    3. When you have a blocked blood vessel in your heart    4. When you get very stressed, and you don’t act like yourself    5. I don’t know 4. Which of these is a symptom of a stroke?    1. Sudden chest pain    2. Sudden shortness of breath    3. Sudden slurred speech or trouble speaking    4. Sudden stomach pain    5. I don’t know 5. Which of these is a symptom of a stroke?    1. Sudden coughing spell    2. Sudden weakness on one side of the body    3. Sudden back pain    4. Sudden fever    5. I don’t know 6. What should you do if you think someone is having a stroke?    1. Drive them to the hospital    2. Tell them to call their doctor    3. Tell them to lie down and rest    4. Call 911 immediately    5. I don’t know 7. What is the *most* important thing you can do to prevent a stroke?    1. Stop drinking coffee    2. Avoid physical activity    3. Control your blood pressure    4. Decrease stress    5. I don’t know 8. Which doctors do I need to see in clinic after I leave the hospital?    1. A primary care doctor    2. A stroke doctor    3. A primary care doctor and a stroke doctor    4. I only need to see the doctor if I have new problem    5. I don’t know 9. How certain are you that you could recognize the symptoms of a stroke?    1. Very certain    2. Somewhat certain    3. Somewhat uncertain    4. Very uncertain 10. How satisfied are you with the stroke education that you received in the hospital?     1. Very satisfied     2. Satisfied     3. Unsatisfied     4. Very unsatisfied |
| --- |
